# Carbonyl-based blue autofluorescence of proteins and amino acids

**DOI:** 10.1371/journal.pone.0176983

**Published:** 2017-05-25

**Authors:** Chamani Niyangoda, Tatiana Miti, Leonid Breydo, Vladimir Uversky, Martin Muschol

**Affiliations:** 1 Department of Physics, University of South Florida, Tampa, Florida, United States of America; 2 Department of Molecular Medicine, USF Health, Tampa, Florida, United States of America; Kermanshah University of Medical Sciences, ISLAMIC REPUBLIC OF IRAN

## Abstract

Intrinsic protein fluorescence is inextricably linked to the near-UV autofluorescence of aromatic amino acids. Here we show that a novel deep-blue autofluorescence (dbAF), previously thought to emerge as a result of protein aggregation, is present at the level of monomeric proteins and even poly- and single amino acids. Just as its aggregation-related counterpart, this autofluorescence does not depend on aromatic residues, can be excited at the long wavelength edge of the UV and emits in the deep blue. Differences in dbAF excitation and emission peaks and intensities from proteins and single amino acids upon changes in solution conditions suggest dbAF’s sensitivity to both the chemical identity and solution environment of amino acids. Autofluorescence comparable to dbAF is emitted by carbonyl-containing organic solvents, but not those lacking the carbonyl group. This implicates the carbonyl double bonds as the likely source for the autofluorescence in all these compounds. Using beta-lactoglobulin and proline, we have measured the molar extinction coefficients and quantum yields for dbAF in the monomeric state. To establish its potential utility in monitoring protein biophysics, we show that dbAF emission undergoes a red-shift comparable in magnitude to tryptophan upon thermal denaturation of lysozyme, and that it is sensitive to quenching by acrylamide. Carbonyl dbAF therefore provides a previously neglected intrinsic optical probe for investigating the structure and dynamics of amino acids, proteins and, by extension, DNA and RNA.

## Introduction

Autofluorescence from proteins is typically equated with the intrinsic UV fluorescence of tryptophan and, to a lesser extent, tyrosine and phenylalanine. [1] The molecular substrate for the intrinsic fluorescence in these three amino acids arises from the delocalized electronic states of their indole and phenyl residues, respectively. The molar absorptivity and high quantum yield, combined with the intrinsic sensitivity of tryptophan fluorescence to its environment, has made tryptophan a widely used optical probe for measuring numerous aspects of protein structure and dynamics. Its applications range from routine determinations of protein concentrations *via* UV absorbance,[2] over monitoring of protein unfolding from conformation-dependent Stokes shifts, [3] to recent investigations of peptide dynamics from tryptophan triplet-state quenching rates. [4, 5] Hence intrinsic protein fluorescence provides a powerful and label-free readout of many aspects of protein properties and their sensitivity to the solution environment.

Recently, a novel visible autofluorescence was observed upon formation of protein crystals, amyloid fibrils and spherulites.[6–9] This autofluorescence is excited at the edge of the long-wavelength UV range (~ 360–380 nm) and emits in the deep blue (~ 450 nm). Findings in our lab suggested that precursors to this aggregation-associated fluorescence might have already existed prior to protein aggregation. Here we present evidence that dbAF can be traced down to an underlying weak intrinsic fluorescence in monomeric proteins, intrinsically disordered polypeptide chains and, most surprisingly, in all single amino acids we looked at. As a first step towards characterizing the optical properties of dbAF we determined the molar extinction coefficients ϵ and quantum yields (QY) of dbAF for the protein beta-lactoglobulin (BLG) and for the amino acid proline (Pro). We furthermore identified the likely molecular substructure underlying this amino acid autofluorescence. Specifically, organic compounds that contain carbonyls, such as formaldehyde and acetone, displayed autofluorescence with spectral characteristics similar to dbAF from amino acids and proteins. At the same time, autofluorescence from closely related compounds without the carbonyl group (methanol, isopropanol) was essentially non-existent. Therefore we propose that carbonyls are the dominant molecular substrate for the observed dbAF from amino acid and proteins.

We further present evidence that dbAF emission from proteins, similar to tryptophan fluorescence, can be used to investigate protein structure. We show that dbAF emission undergoes a red-shift upon thermal denaturation of lysozyme and that dbAF fluorescence can be quenched by the fluorescence quencher acrylamide. Our results indicate that dbAF represents a previously unexplored and ubiquitous intrinsic autofluorescence that can provide new insights into a variety of biophysical and biochemical processes in amino acid, polypeptide and protein biophysics and biochemistry.

## Materials and methods

### Proteins and chemicals

Two times recrystallized and lyophilized hen egg white lysozyme (hewL) was purchased from Worthington Biochemicals. We have previously characterized the purity and monomeric character of this stock material when following proper preparation. [10] To exclude presence of small fluorophores, we dissolved lyophilized hewL at 40 mg/ml in deionized water, placed it into a 6–8 kD MWCO dialysis tube, and dialyzed against 2 L of deionized water for two days with four changes of water. Lyophilized β-lactoglobulin (BLG), Poly-L-lysine hydrobromide (PLK), L-lysine, poly-L-glutamic acid (PLE), L-glutamic acid, L-cysteine, glycine, quinine sulfate, isopropanol and acetone were obtained from Sigma-Aldrich and used without further purification. Bovine serum albumin (BSA) and L-tryptophan were from MP Biomedicals. Remaining amino acids were purchased from Anaspec. All other chemicals were purchased from Fisher Scientific. Solvents and chemicals were reagent grade or better. Solutions were prepared using 18 MΩ water from a reverse osmosis unit (Barnstead E-pure).

### Preparation of samples and solutions

Proteins and polyamino acids were dissolved in pH 7 buffer (20 mM HEPES, no salt or 50 mM HEPES and 50 mM NaCl, respectively) and typically filtered through 220 nm and 50 nm pore size filters. Protein concentrations were determined from UV absorption at 280 nm using the appropriate UV absorption coefficient. Amino acids were dissolved in either pH 2 potassium phosphate buffer or pH 7 HEPES buffer and concentrations were determined from the weight to solvent volume ratios. Amyloid fibrils of hen egg-white lysozyme (hewL) were grown by incubating 20 mg/ml of hewL in 25 mM phosphate buffer at pH 2, 100 mM NaCl at 52°C for 3–5 days. Details of amyloid fibril growth and characterization are provided elsewhere. [11, 12] For determination of the dbAF increase upon hewL fibril formation, amyloid fibrils were separated from their monomeric background *via* three rounds of centrifugations (24 hours, 16,000 rpm) and resuspensions of the fibril pellet in buffer. Based on the monomer concentration in the supernatant vs. the total protein concentration in the fibril pellet after the 3^rd^ round, the residual monomer content in the purified fibril suspension was estimated to be 1%.

### Fluorescence spectroscopy

A FluoroMax-4 spectrofluorometer was used for all fluorescence measurements. Unless specifically stated, instrument settings were kept constant to permit comparison of relative intensities across samples. DbAF was typically excited near 370 nm and emission spectra were measured between 400 and 600 nm. With the exception of the thermal denaturation data, dbAF spectra were collected at room temperature. Buffer/salt solutions or deionized water were routinely checked for background fluorescence that might interfere with dbAF signals. Tryptophan fluorescence during thermal denaturation was excited at 280 nm using hewL concentrations of 5 μg/ml. Fluorescence quenching data were collected using 2.5 mg/ml BLG at pH 2 and 25°C at the indicated concentrations of acrylamide. For each BLG/acrylamide mixture, background spectra without BLG but at the same acrylamide concentration were collected. The buffer spectrum (dominated by the water Raman peak) was measured and subtracted from both sets of spectra before subtracting acrylamide from BLG/acrylamide spectra. The Stern-Volmer constant was determined from a plot of the peak dbAF fluorescence of BLG before adding acrylamide (dbAF_0_) over its corresponding values in the presence of the stated concentrations of acrylamide (dbAF), after correcting these spectra for their buffer and acrylamide background.

### Epifluorescence images of amino acid crystals

Microscope fluorescence images were acquired on an inverted microscope (Olympus IX-70) using a 40x, 1.15 NA water immersion objective (Olympus UApo/340) and a 1.5x tube lens. Images were recorded with a high-sensitivity camera (Andor Ixon, DV-885K) cooled to -60°C with preamplifier gain set to 3.6x, but without EM gain. Epifluorescence illumination was provided by a 385 nm LED (Thorlabs M385L2, 750 mA drive current) passing through a standard DAPI filter cube (Chroma 31000: AT350/50 ex; 400 dclp dichoroic; D460/50 em). Integration times for epifluorescence images varied from 0.5 s to 20 s, and are indicated in the figure caption for S2 Fig in S1 File. Fluorescence images were processed to provide intensity distributions and contrast suitable for visualization. For quantification of relative dbAF intensities from different crystals the average fluorescence intensities was measured over a large “region of interest”, thereby integrating over the noticeable variations in dbAF intensities. Background fluorescence measured over a comparable area off the crystals was subtracted and the resulting difference values were divided by the exposure time used to collect the images. The resulting relative dbAF intensities for amino acid crystals were plotted against the peak dbAF intensity of the same amino acid measured in solution.

### Molar extinction coefficient and quantum yield determination

Absorption spectra were recorded on a Lambda 950 UV/vis Spectrometer using 10 mm pathlength quartz cuvettes. Beer’s Law relates absorbance A, molar extinction coefficient ε, molar concentration c and the pathlength l of the cuvette *via* A = ε c l. Molar extinction coefficients of BLG (18.4 kD) and proline (115.1 Da) were determined from linear fits to the absorbance A at 350 (Pro) or 357 nm (BLG) vs. their corresponding molar concentrations. Quantum yields QY for dbAF of proline (Anaspec) and BLG (Sigma) were determined *via* comparison of their dbAF emission amplitudes with the quantum yield standard quinine sulfate. [12] Quinine sulfate was dissolved in 0.1 M sulfuric acid and its fluorescence measured at concentrations between 1 to 5 μg/ml. Quinine sulfate emission was excited at 350 nm, while BLG and Proline dbAF were excited at 357 and 350 nm, respectively. Quinine sulfate fluorescence intensities were corrected for the use of an ND1 neutral-density UV filter in the excitation path and a 395 LP Schott filter in the emission path with a measured attenuation of 88% and 21%, respectively. Solvent backgrounds were subtracted from all fluorescence emission spectra. Peak fluorescence emissions were plotted against absorbance at the optimal excitation wavelength for a series of different concentrations of quinine sulfate, BLG and proline. These plots were linear, as required to exclude inner filtering, aggregation or other systematic measurement errors. The slopes S_X_ (were the subscript X stands for either proline or BLG) of the fluorescence emission vs. absorbance plots and for the quinine sulfate standard (S_QS_) were determined. The corresponding quantum yield QY_X_ was then determined from the ratio of the slopes S_X_/ S_QS_, multiplied by the quantum yield QY_QS_ of quinine sulfate of 0.54 [13], i.e.

$${QY}_{x}={QY}_{QS}(S_{x}/S_{QS})$$ (1)

For additional details we refer the reader to ref. [13] or the many other reviews on this topic.

## Results

### Deep-blue autofluorescence (dbAF) in globular proteins

**Fig 1:**
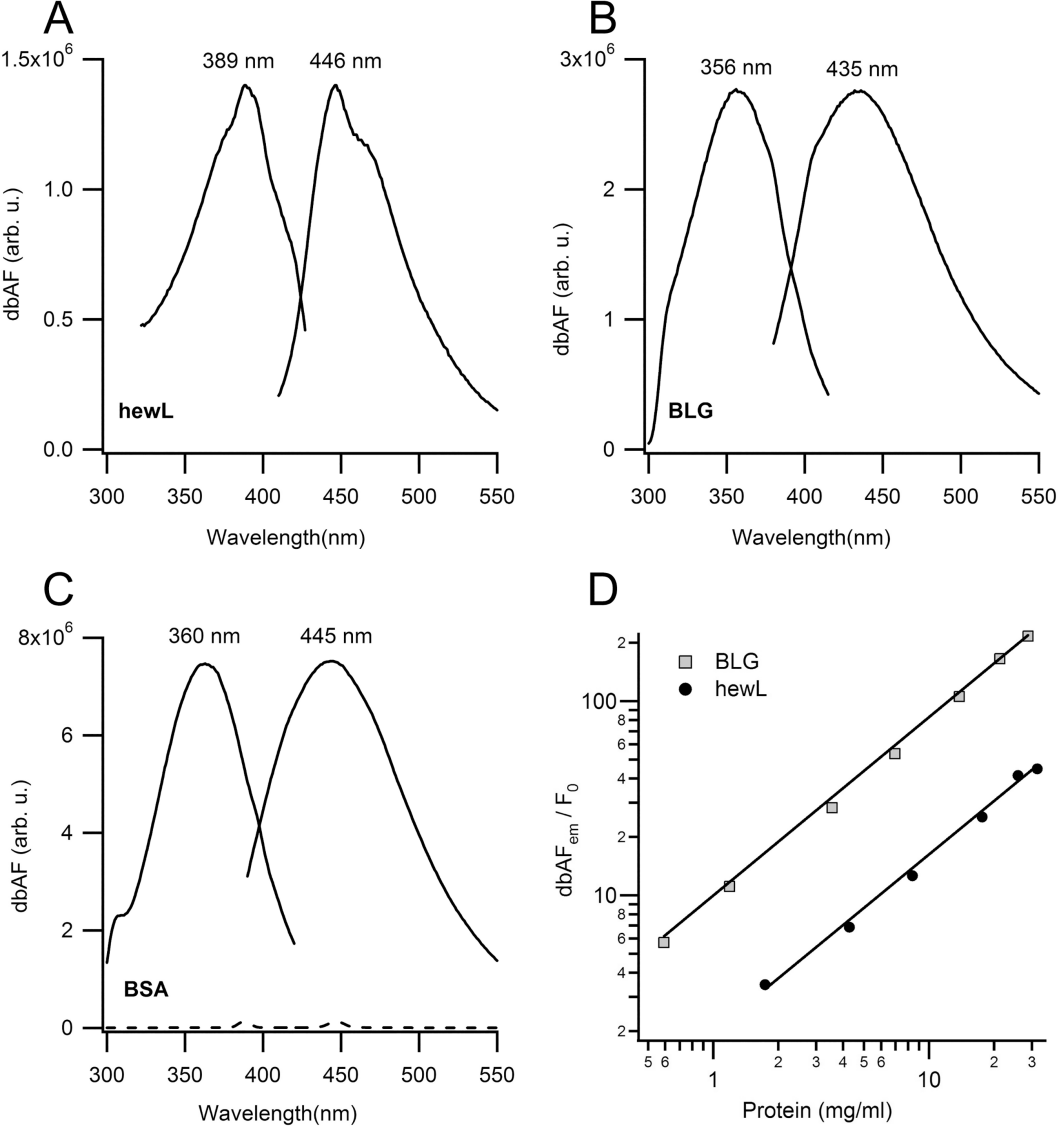
Deep-blue autofluorescence (dbAF) spectra of globular proteins. Fluorescence excitation and emission spectra for deep-blue autofluorescence (dbAF) from (**A**) hen egg-white lysozyme (hewL) (**B**) β-lactoglobulin (BLG) and (**C**) bovine serum albumin (BSA), all at 50 mg/ml. The dashed line near the bottom in (C) is the buffer background spectrum with its associated Raman peak. DbAF peak positions reported for the weaker hewL emissions are likely distorted by contribution from the buffer Raman peaks we couldn’t fully correct for. Spectra in (A-C) used identical spectrofluorimeter settings and all spectra were collected at pH 7 (20 mM HEPES). (**D**) Log-log plot of the concentrations dependence of dbAF peak emission for BLG and hewL, expressed as multiples above their buffer background F_0_. The solid lines are fits with a linear function through the data.

Looking for the previously reported fluorescence signal upon amyloid fibril formation by hen egg-white lysozyme (hewL) [7] we noticed a weak intrinsic fluorescence was already present prior to the onset of aggregation. We therefore investigated whether this weak fluorescence was a feature common to proteins other than lysozyme and, if so, what the underlying molecular substrate of this signal might be. As shown in Fig 1, the fluorescence emission we had observed with hewL monomers (Fig 1A) was even more prominent in solutions of β-lactoglobulin (BLG, Fig 1B) and bovine serum albumin (BSA; Fig 1C). The fluorescence excitation and emission spectra for all three proteins had similar spectral characteristics, with excitation maxima ranging from 355 nm to 390 nm and peak emission near 440 nm. Protein-specific differences included the intensity of dbAF for different proteins, the wavelengths for peak emission and peak excitation as well as the overall width of excitation and emission spectra. To exclude potential measurement artifacts from e.g. second-order diffraction peaks of the fluorometer gratings or from Rayleigh light scattering off proteins in these highly concentrated solutions we confirmed that high-Q optical filters placed in both the excitation (360/20 nm) and emission (395 nm lp) path preserved dbAF emissions. We also checked for background fluorescence from all buffer solutions used for measurements (see e.g. Fig 1C) to confirm that the fluorescence arose from the proteins added.

### Does deep-blue autofluorescence (dbAF) arise from protein monomers?

We took several precautions to assure that the dbAF emissions in Fig 1 did indeed arise from protein monomers. To exclude potential contamination by small fluorophores, we extensively dialyzed the hewL stock and compared dbAF spectra of concentration-matched samples before and after dialysis. Their spectra remained within a few percent of each other (S1 Fig in S1 File). As noted above, dbAF emission has been previously associated with the formation of protein aggregates, most prominently amyloid fibrils. To minimize aggregation under our measurement conditions, all fluorescence measurements, with the exception of the thermal denaturation data discussed below, were performed at room temperature. This eliminated the risk of forming “prefibrillar aggregates” since fibril growth by folded proteins, at a minimum, requires heating past the onset of thermal denaturation (see e.g. ref [14]). In addition, we used low ionic strength solutions that tend to maximize protein solubility. For example hewL at pH 7 and ionic strength at 50 mM is soluble to at least 200 mg/ml, a value that increases even further at pH 2 used for measurements in fibril growth buffers. [15] Guptasarma et al. [16] had previously identified comparable dbAF emissions from γ-B crystallin which has an even higher intrinsic solubility. Using dynamic light scattering, we confirmed the absence of large non-equilibrium clusters that can be present in lyophilized proteins. [10] To address the potential formation of small oligomers, we mapped out the concentration dependence of dbAF emissions for both BLG and hewL. As shown in Fig 1D, we could detect dbAF emissions five times above background at concentrations as low as 0.5 mg/ml for BLG and three times for 2 mg/ml of hewL. Equally important, the strictly linear increase of dbAF over as many as two orders of magnitude in protein concentration argues against oligomer formation. Oligomer binding equilibria result in sublinear increases in oligomer populations, and their associated dbAF emission, with monomer concentration.

**Fig 2:**
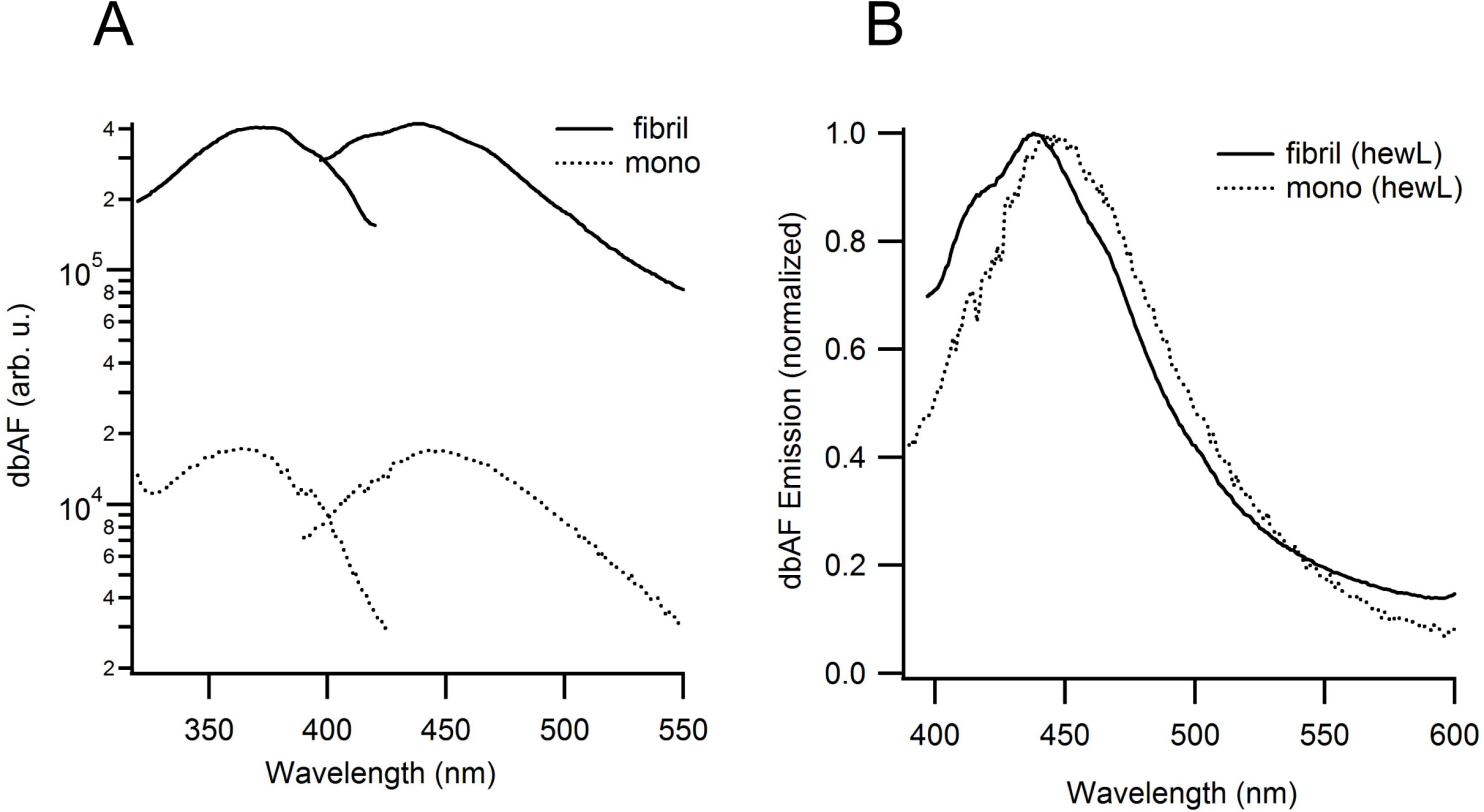
Comparing dbAF emission from lysozyme fibrils and monomers. **(A)** Quantitative comparison of dbAF emission spectra from hewL monomers and purified hewL fibrils in pH 2 buffer, 100 mM salt measured at room temperature. Spectra are shown on a semi-log scale to facilitate comparison of relative enhancement. The control monomer spectrum was taken with hewL stock freshly dissolved in fibril growth buffer and concentration-matched to the pure fibril sample. **(B)** Superposition of dbAF emission spectra for hewL amyloid fibrils and monomers, measured at room temperature in fibril growth buffer. Spectral amplitudes were normalized to their peak emission values.

To determine whether minor populations of small protein aggregates, if present, could explain the observed dbAF emission, we determined dbAF intensities from fully aggregated hewL amyloid fibrils. HewL fibrils, purified to 99% by three consecutive centrifugation/resuspension steps, generated a significant but still moderate 25-fold increase in dbAF emission over their equivalent monomeric background (Fig 2A). We therefore estimate that 4% of monomers would have to be at least dimerized to account for the observed dbAF emission from monomeric solutions. When considering the lower number of intermolecular bonds in dimers vs. fibrils, which are believed to underlie fibrillar dbAF emission, the fraction of required preexisting aggregates to account for dbAF emission from monomeric solutions would increase even further. We have shown previously that static light scattering from the same hewL stock, over the same range of concentrations, extrapolates to the proper molecular weight for lysozyme monomers (14.3 kDa) and dynamic light scattering yielded polydispersity indices as low as 0.15 or less. [17] We therefore estimate that our solutions could contain at most one percent of protein dimers, with even lower values when allowing for larger oligomers. For the above reasons, preexisting aggregates can’t account for the dbAF emissions from our monomeric hewL solutions.

In addition, the same type of dbAF signal is emitted from both BLG and BSA, which similarly would have to be contaminated by pre-existing aggregates. For BLG, in particular, the low levels of protein concentrations showing significant dbAF emissions make this highly unlikely. While sparse, there are at least two prior reports of dbAF emission from monomeric proteins. The earliest mention is by Kallman et al. who recorded dbAF emission from collagen. [18] More recently, Guptasarma collected similar dbAF spectra from both γ-B crystallin and lysozyme. [16] Both agree well with our observations. Therefore, there is mounting evidence from multiple laboratories that monomeric proteins of various types and structures emit a deep-blue autofluorescence. Fig 2B shows the superposition of dbAF spectra from hewL fibrils vs. those from monomers. While there are subtle differences, the obvious similarity of fibrillar vs. monomeric excitation/emission spectra implies that the molecular moiety underlying fibrillar dbAF is already present in monomeric proteins.

### DbAF is present in poly-amino acids and single amino acids

**Fig 3:**
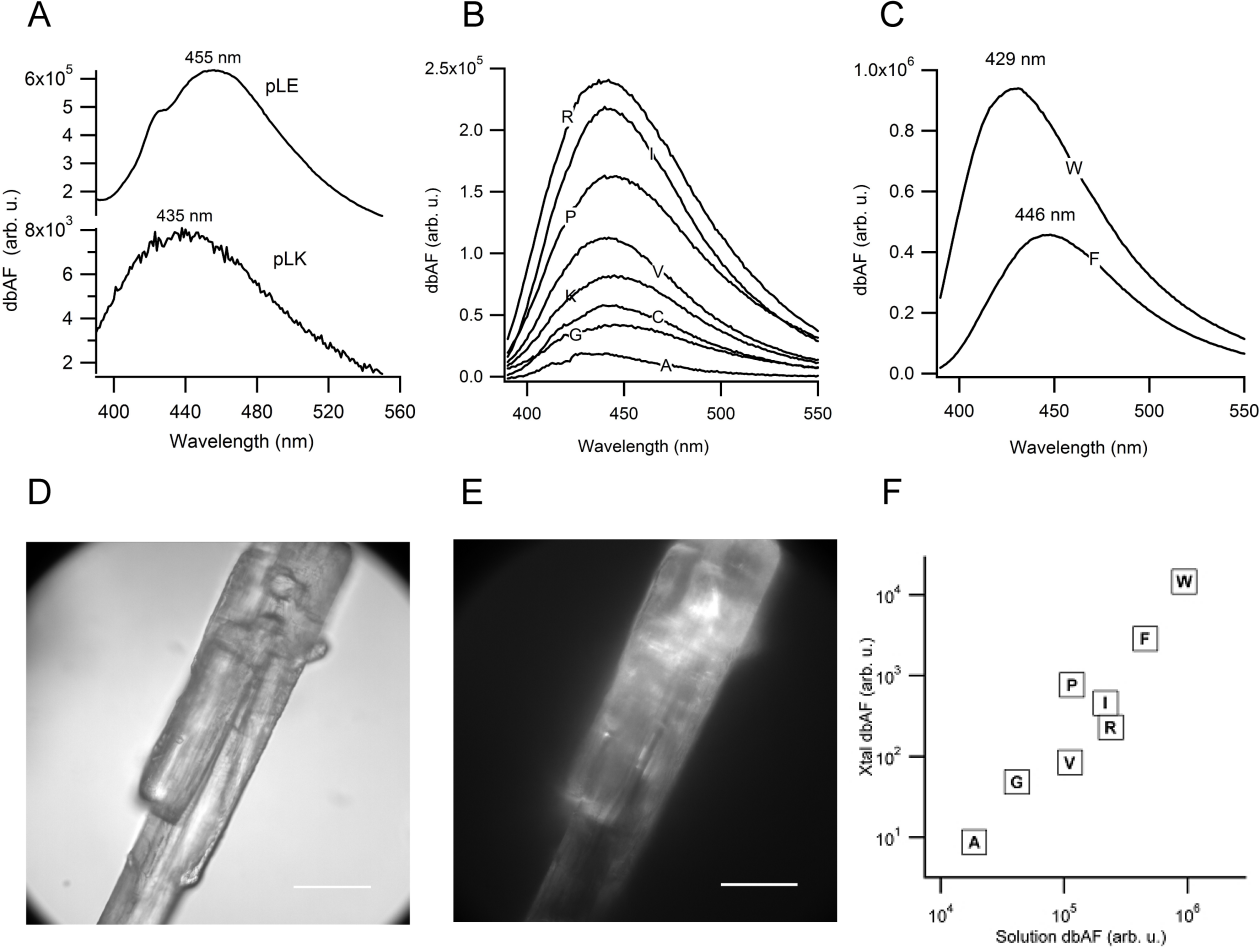
DbAF from polyamino acids and individual amino acids. DbAF emission spectra (370 nm ex.) for **(A)** poly-l-lysine (PLK) and poly-l-glutamic acid (PLE) at 10 mg/ml each and at pH 7 (50 mM HEPES). **(B)** DbAF emissions from eight different non-aromatic amino acids (indicated by their standard single-letter code) at 20 mg/ml each and at pH 2 (25 mM phosphate buffer). All spectra in (B) were buffer subtracted. **(C)** Same as (B) but for the aromatic amino acids tryptophan (W) and phenylalanine (F), which display much larger dbAF (see scale in B vs. C). Data for the spectra in Fig 3 (B) and (C) are available at https://figshare.com/s/f0b53a0fe5cd23561b18. **(D)** Brightfield image of proline crystal and corresponding **(E)** epifluorescence image with standard DAPI illumination/filter set (additional images of eight different amino acid crystals are shown in S2 Fig in S1 File). **(F)** Correlation of average dbAF intensities from amino acid crystals vs. corresponding peak intensities of their dbAF solution spectra. Amino acids are indicated by their standard single-letter code.

The above results raise the question what the identity of the moiety underlying dbAF could be? The emission of dbAF from amyloid fibrils and protein aggregates has been associated with the formation of intermolecular hydrogen bonds as source of electron delocalization.[7, 9, 19] The presence of dbAF from monomeric proteins and their intramolecular hydrogen bonds would be consistent with this interpretation. However, we wanted to explore how far below the structure of folded proteins one could go while preserving dbAF emission. As a first step towards dissecting the origin of dbAF, we explored whether a native fold or well-defined secondary structure elements such as α-helices or β-sheets were a prerequisite for dbAF emission. The polyamino acids poly-l-glutamic acid (PLE) and poly-l-lysine (PLK) are known for assuming random-coil conformations at neutral pH. [20, 21] As shown in Fig 3A, both PLE and PLK emitted dbAF, albeit at different peak emission wavelength and with significantly different amplitudes at comparable mass concentrations. Hence, dbAF persists in polypeptides and, therefore, doesn’t require either a native fold or a well-defined secondary structure but is intrinsic to polypeptide chains themselves. Perhaps more so than with the intact proteins, the intensities and emission peaks of dbAF from polypeptides displayed differences that could be exploited in future applications. The autofluorescence from these simple polyamino acids lacking any aromatic residues reinforces observations with amyloid fibrils from elastin-like pentapeptides that dbAF is distinct from the “conventional” protein autofluorescence arising from aromatic amino acids. [1, 6]

The above data do not address, though, what molecular bonds and associated electronic band structures sustain dbAF from polyamino acids and proteins. One potential source could be the weak delocalization of electrons induced by the peptide bonds of polypeptides and proteins. Conjugated systems with alternating double and single bonds are known to support fluorescence of synthetic polymers and aromatic compounds in the visible range. [22] The partial double-bond character of peptide bonds might therefore provide the substrate for dbAF emissions. If so, the formation of peptide bonds upon amino acid polymerization would be a prerequisite for dbAF and, conversely, individual amino acids should lack dbAF. Yet, every one of ten different amino acids we investigated, which included charged, hydrophobic (Fig 3B) and aromatic amino acids (Fig 3C), showed weak but clearly detectable dbAF emissions of varying intensities and with emission peaks near 440 nm. DbAF emission from the two aromatic amino acids tryptophan and phenylalanine was noticeably higher than from non-aromatic amino acids and the emission peak of tryptophan blue-shifted towards 430 nm. We also find that crystals of the amino acids emit bright dbAF when visualized in epifluorescence using standard 4',6-diamidino-2-phenylindole (DAPI) fluorescence filters. Fig 3 shows the resulting brightfield (Fig 3D) and epifluorescence (Fig 3E) images for a proline crystal, with images for seven additional amino acid crystals provided in S2 Fig The significant variation in dbAF fluorescence intensity emitted from different amino acids in solution (Fig 3B and 3C) strongly correlates with the relative dbAF fluorescence emitted from the corresponding amino acid crystals (Fig 3F). The prominent variation in dbAF emission intensities and wavelengths with amino acid identity, irrespective whether measured in solution or in the solid state, suggests that dbAF emission is sensitive to the amino acids chemical structure (residue), irrespective of the specific environment they are in.

### Carbonyls are the likely source of dbAF autofluorescence

**Fig 4:**
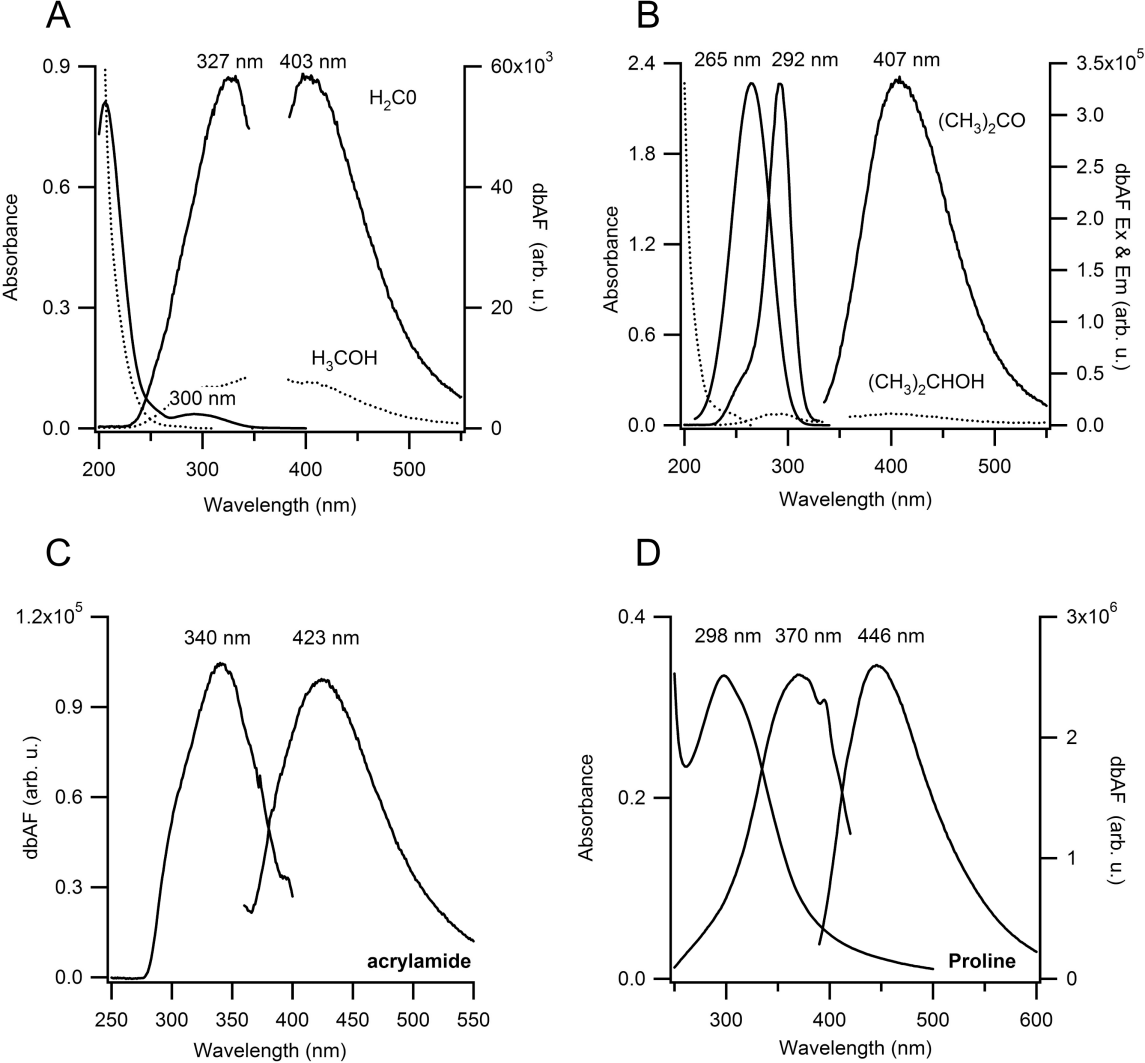
DBAF of organic solvents with and without carbonyl groups. Absorption, fluorescence excitation and emission spectra of **(A)** formaldehyde (CH_2_O) vs. methanol (CH_3_OH) at 10 M concentrations. **(B)** 170 mM acetone (CH_3_)_2_CO vs. undiluted (13.1 M) isopropanol (CH_3_)_2_CHOH and **(C)** Fluorescence excitation and emission spectra of 700 mM acrylamide (C_5_H_8_NO) **(D)** Absorption, excitation and emission spectra from 1.3 M proline at pH 7 (20 mM HEPES), buffer subtracted. Excitation spectrum of acetone was distorted due to strong inner filtering from UV absorption. Gaps in spectra in (A) are due to removal of large solvent Raman peaks. Large water Raman peak was subtracted in (C).

The above observations indicate that the molecular structure(s) that give rise to dbAF are already present at the level of individual amino acids. One potential candidate is the C = O carbonyl double bond present in all amino acids. To explore this possibility we looked for dbAF fluorescence from simple organic compounds with carbonyls: formaldehyde (H_2_CO), acetone ((CH_3_)_2_CO) and acrylamide (C_3_H_5_NO). All three compounds displayed noticeable autofluorescence. The excitation and emission peaks for acetone and formaldehyde were blue-shifted between 40–70 nm compared to those of amino acids and proteins (Fig 4A and 4B). All of them also lack the carboxyl group present in amino acids, suggesting that the latter is not essential for dbAF emission. Addition of the amide group in acrylamide shifted both the excitation and emission spectra noticeably towards the visible range (Fig 4C), with values that approach those of amino acids and proteins (Figs 1 and 4D). This suggests that the addition of the amide group near the carbonyl has the ability to shift fluorescence properties of the carbonyl groups significantly. At a minimum, the above data indicate that small organic molecules with carbonyl groups, at high concentrations, are capable of fluorescence emission in the deep blue. In addition, the details of the molecular structure surrounding the carbonyls clearly affected fluorescence intensities and associated spectral properties. This is likely to contribute to the large difference in emission from formaldehyde vs. acetone, particularly when accounting for their difference in molar concentrations (170 mM for acetone vs. 10 M for formaldehyde). To confirm the important role of carbonyls as the structural element underlying this autofluorescence, we looked for intrinsic fluorescence from methanol (H_3_COH) and isopropanol (CH_3_)_2_CHOH, in which the carbonyl in formaldehyde and acetone is replaced by a hydroxyl group. This nearly abolished autofluorescence from either compound (Fig 4A and 4B). These observations indicate that one should *expect* to find intrinsic fluorescence in the deep blue from any molecule containing carbonyl groups, including proteins and amino acids. At the same time, the residues surrounding the carbonyl groups in these small organic molecules have significant effects on the absorption and fluorescence excitation spectra and their spectral overlap. This matches well with the variable dbAF intensities from amino acids, as shown in Fig 3.

Interestingly, the intrinsic fluorescence of acetone and formaldehyde are well established and have been utilized for some time for remote sensing in nonreactive and reactive flows. [23, 24] Corresponding quantum-mechanical calculations of acetone absorbance and fluorescence properties, particularly in solution, have become available recently, as well. [25, 26] Autofluorescence of polyacrylamide gels with similar characteristics as those in Fig 4C have also been noted. [27] We are not aware of any comparable theoretical or experimental reports of dbAF emission from amino acids without aromatic residues. Following our identification of carbonyls, we found one reference 50 years back implicating carbonyls as source of collagen fluorescence, but without proof of its origin at the single amino acid level or any detailed physiochemical characterization.[18] This earlier observation was apparently discredited as artifact of UV-induced photoproducts of collagen that can be phosphorescent.[28] As we point out above, the wide variety of proteins, polypeptides and single amino acids exhibiting this fluorescence, and its close association with both carbonyl double bonds and its congruence with aggregation-induced fluorescence suggest that this earlier observation was likely correct.

The spectra in Fig 4 reveal another feature of dbAF that seems to be shared by proteins, amino acids and organic solvents with carbonyls: there is a noticeable offset between the optimal wavelength for photon absorption vs. fluorescence excitation, as shown here for proline (Fig 4D) formaldehyde (Fig 4A) and acetone (Fig 4B). This suggests that the most favorable wavelength for photon absorption excites electrons into states above the lowest excited state and, therefore, might undergo non-radiative energy loss prior to emission. Hence, fluorescence emission will depend on the spectral overlap between the fluorescence excitation spectra with the molar absorptivity. In the case of proteins, the weak absorbance associated with dbAF does not result in a distinct peak but seems buried underneath residual absorbance from aromatic residues (see S3 Fig in S1 File). Such displacements between maximal absorbance and optimal fluorescence excitation are common for other small molecules, such as the indole moiety of tryptophan. [29] At the same time, strong fluorophores such as quinine only have a minimal if any shift between their (fluorescence-associated) absorbance and fluorescence excitation. Hence, this spectral feature of dbAF provides some additional support for carbonyls as the shared molecular substrate for dbAF across these otherwise very different molecules.

### Quantum-yield and molecular extinction coefficients of dbAF

As step toward further characterization of the photophysical properties of dbAF from proteins and amino acids, we determined the molar extinction coefficient and quantum yield for proline and for BLG. As reference standard for quantum yield in the wavelength range near dbAF we used quinine sulfate with a quantum yield of 0.54 (for details of sample preparation and measurements see Materials and Methods). [13] To account for the shift in maximal absorption vs. excitation of dbAF spectra in the determinations of quantum yields, we used the molar extinction coefficients ε at the peak of the fluorescence excitation spectrum which, for proline in pH 7 buffer, occurs at 350 nm and for BLG occurs at 357 nm. Plots of fluorescence emission vs absorbance for BLG and proline used to extract the slopes S_BLG_ and S_Pro_ in Eq 1 above are shown in S3 Fig in S1 File. The dbAF quantum yields QY we obtained for proline and BLG at pH 7, listed in Table 1, are very similar and in a range only about one order of magnitude below common fluorophores.

**Table 1:** Molar extinction coefficient ε and quantum yield (QY) for dbAF from BLG and proline at pH 7.

	ε (mol^-1^cm^-1^)	QY
beta-lactoglobulin (BLG)	43.3 (357 nm)	0.011
Proline (Pro)	0.036 (350 nm)	0.012

In contrast, the molar extinction coefficient of both BLG and proline are significantly smaller than for typical fluorophores (e.g. tryptophans molar extinction coefficient is 5,690 M^-1^cm^-1^). Yet, the small molar extinction coefficients and the fluorescence emission in the visible would be compatible with a nonbonding to antibonding n-π* transition. Guptasarma [16] reported that dbAF excitation in γ-B crystallin was associated with a CD signal. While this author guardedly associated the chirality with hydrogen bonding within the protein’s secondary structure, it might instead arise from the chirality of n-π* transitions in carbonyl compounds. [30] Such n-π* transition have also been implicated in the fluorescence from various aldehydes. [31]

There is a noticeable difference in the molar extinction coefficients of BLG and proline, even after multiplying ε for proline by 162, i.e. the number of amino acids in BLG. However, uncertainty in the absolute values of amino acid concentrations and variability in dbAF emission intensities might contribute to the observed discrepancies. Additional considerations compel us to classify the EC/QY measurements as estimates. These include the difficulty in separating the fluorescence-associated absorbance from overlap with other non-dbAF related absorbance, as well as the strong sensitivity of our values to subtle changes in solution conditions (pH, salt, temperature). A detailed investigation of the relation between the dbAF of carbonyls and its modulation by the vicinity to different amino acid residues, its sensitivity to neighboring residues, its relation to the molecular structure of the protein and the origin of its aggregation-related augmentation are well beyond the scope of the current paper.

### Potential utility of DbAF for studying protein structure and function

**Fig 5:**
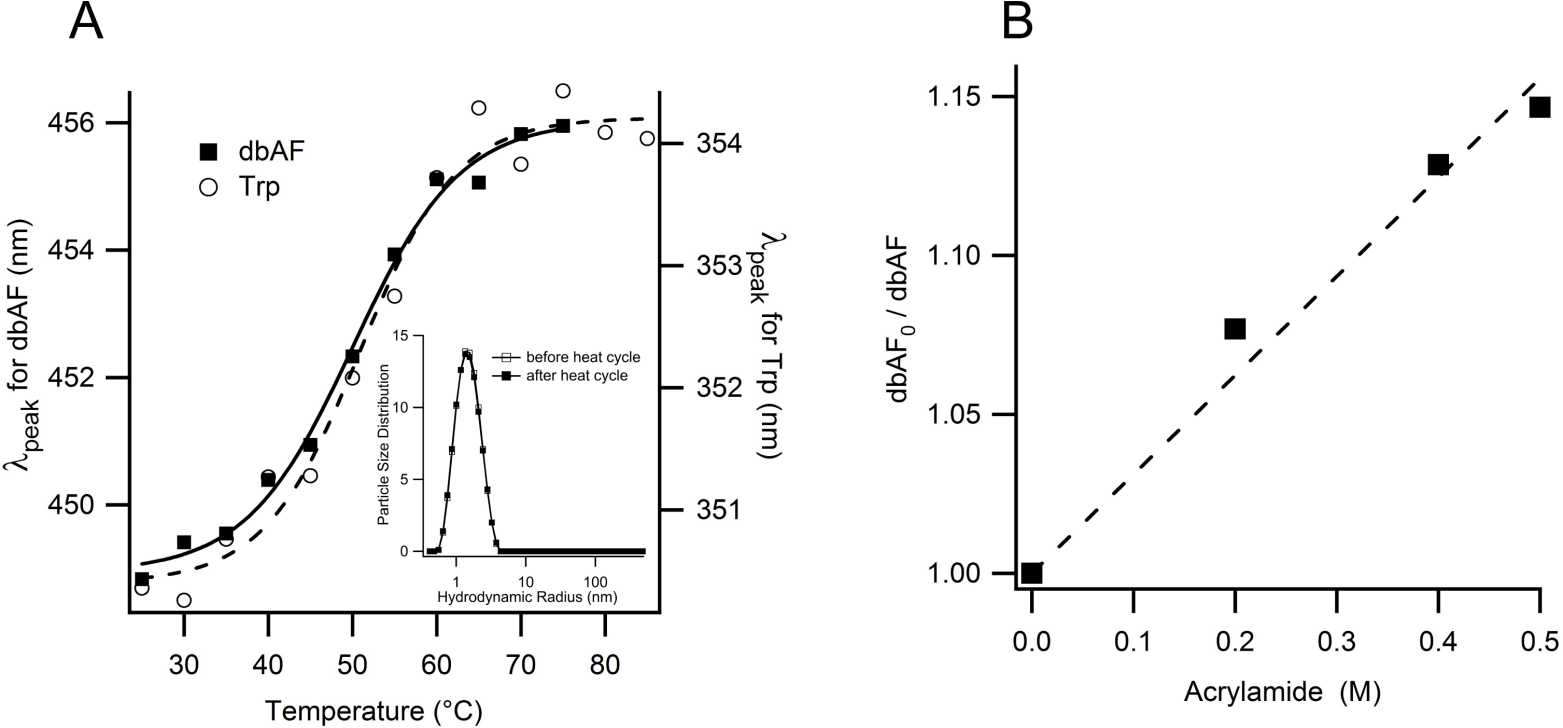
dbAF emission tracks lysozyme denaturation, is sensitive to quenching. **(A)** The peak wavelength λ_max_ of the fluorescence emission for dbAF (■, left axis) and Trp (○, right axis) during thermal denaturation of hewL at pH 2. Lines are sigmoidal fits through the dbAF (―) and Trp (- -) data, respectively. The insert shows the particle size distribution derived from dynamic light scattering of the dbAF solution prior to and after heat cycling. **(B)** Stern-Volmer plot for quenching of dbAF emission from BLG (2.5 mg/ml) by increasing concentrations of acrylamide. Here, dbAF_0_ represents the dbAF emission from BLG without acrylamide. DbAF from acrylamide at a given concentration was subtracted from dbAF values for BLG/acrylamide mixtures.

To explore the utility of dbAF as intrinsic probe of protein properties we investigated whether dbAF could substitute for tryptophan fluorescence for tracking thermal protein unfolding. Upon denaturation of natively folded proteins, tryptophans in the interior of proteins become exposed to water which induces a red-shift in their florescence emission wavelength. [1] Fig 5A documents this red-shift of tryptophan fluorescence during the cooperative unfolding of lysozyme at pH 2. Superimposed to the red shift in tryptophan fluorescence is the peak dbAF emission wavelength recorded under the same conditions. As shown, dbAF emission undergoes a red-shift of near identical magnitude to that of tryptophan upon lysozyme unfolding. However, dbAF detection required relative high hewL concentrations, which could have induced aggregation and could have confounded any dbAF changes. By comparing both light scattering intensities (within noise, 0.2% difference) and particle size distributions of hewL solutions prior to and following thermal denaturation (see insert, Fig 5A) we confirmed that the thermal denaturation cycle was short enough to avoid any discernible aggregation. Hence, similar to intrinsic Trp fluorescence, the energetics of the excited states underlying dbAF are sensitive to the protein’s conformation. Our data can’t distinguish whether the observed dbAF red-shift results directly from changes in protein conformation or, alternatively, responds to solvent or electrostatic effects on dbAF that are a consequence of protein unfolding. Guptasarma [16] had previously reported an increase in dbAF emission upon chemical denaturation of lysozyme, but without discernible red-shift. This difference in dbAF response to lysozyme denaturation might arise from differences of solvent effects on dbAF during chemical vs. thermal denaturation, and from differences in solution pH. As additional confirmation of the fluorescence properties and potential applications of dbAF, we established that dbAF fluorescence is quenched, albeit weakly, by acrylamide. Care must be taken to subtract acrylamide spectra at each concentration from protein spectra due to the presence of dbAF-like fluorescence in acrylamide, as well. A Stern-Volmer plot of the data yields a quenching coefficient of K_SV_ = 0.3 M^-1^ (see Fig 5B).

## Discussion

Our investigation implicates carbonyls as the likely source of a weak and previously overlooked autofluorescence from individual amino acids and proteins in the visible range. Our observations with multiple amino acids, several polyamino acids and different proteins from various suppliers extend earlier findings with individual proteins [16, 18], protein crystals [9, 32], polypeptide spherulites [8] and amyloid fibrils [6, 7]. When including not just inter- and intramolecular hydrogen bonding but allowing for hydrogen-bonding with water, our observations are for the most part consistent with previously suggested models of dbAF. At the same time, our measurement do not specifically address the role of hydrogen bonding. The fluorescence emission from other carbonyl-containing compounds (formaldehyde, acetone, acrylamide) suggest that carbonyls could sustain dbAF emission even in the absence of hydrogen bonding. The strong dependence of dbAF intensities with the amino acid identity, irrespective of whether recorded in solution or in the crystalline phase, further suggests that amino acid residues play an important role in modulating dbAF intensities. We can’t exclude, though, that some of the emissions described here are due to phosphorescence instead of fluorescence. For example there are reports of blue phosphorescence from BSA in powder form. [33] The same authors also described phosphorescence emissions similar to those of BSA from the solid but not solution states of carboxyl-rich starch and cellulose.

One intriguing question is why this intrinsic fluorescence from amino acids and proteins has essentially remained unnoticed or unexploited. We are not aware of prior theoretical work suggesting the existence of carbonyl-based fluorescence from individual proteins or amino acids even though carbonyl fluorescence from organic solvents is well established. Among potential reasons for this lack for recognition of dbAF in the protein community might be the weak molar extinction coefficient for dbAF excitation, the blue-shift of its absorption spectrum compared to its fluorescence excitation spectrum and the lack of a discernible shoulder in the absorption spectrum at the optimal excitation wavelength for proteins. The original findings by Kallman et al. of dbAF in collagen were believed to be contaminated by photochemically induced fluorescent byproducts of collagen. [18] It is worth noting that this investigation into potential dbAF from proteins was prompted only by prior reports of significant dbAF from amyloid fibrils. [6] Lifetimes in the nanosecond range established that fibrillar dbAF, measured from amyloid fibrils, resulted from a resonant fluorescence emission instead of some inelastic scattering process. [7] The strong similarities of dbAF spectra from hewL fibrils and monomers (Fig 2B) bolster our hypothesis that the previously identified dbAF from protein aggregates and dbAF from monomeric proteins and amino acids detailed here both arise from their shared carbonyl moieties.

The presence of carbonyl autofluorescence in proteins opens up the possibilities for a wide range of novel applications for studying protein structure and dynamics—particularly at biologically relevant high protein concentrations often present in cellular environments. We have already shown that dbAF emission underwent a prominent red-shift upon thermal denaturation of lysozyme, similar to the well-known behavior of tryptophan fluorescence (Fig 5A). Improved understanding of the sensitivity of dbAF signals to protein structure, state of aggregation, chemical modifications, and various solvent effects is likely to significantly expand the utility of this novel autofluorescence. Our measurements of extinction coefficients and quantum yields, while preliminary, are a first step towards identifying the electronic transitions underlying dbAF. There are clearly many questions regarding the specific role of carbonyls in dbAF, and how amino acid structure, protein structure, and the extent of hydrogen bonding (in aggregates and in solution) alter dbAF properties. We hope that these results will inspire band structure calculations of amino acids underlying dbAF, thereby providing a solid theoretical understanding of amino acid fluorescence.

## Supporting information

S1 File Carbonyl-based blue autofluorescence of proteins and amino acids. Contains supporting information and associated S1 Fig through S3.
(DOCX)
