# Supplementary material for: Carbonyl-based blue autofluorescence of proteins and amino acids
Source: PLoS One. 2017 May 25;12(5):e0176983. doi: 10.1371/journal.pone.0176983 (PMC5444599; doi:10.1371/journal.pone.0176983)
Supplement: S1 File — Contains supporting information and associated S1 Fig through S3. (DOCX) [file pone.0176983.s001.docx]

**Carbonyl-Based Blue Autofluorescence of Proteins and Amino Acids**

**Supporting Information**

Chamani Niyangoda^1^, Tatiana Miti^1^, Leonid Breydo^2^, Vladimir Uversky^2^, and Martin Muschol^1*^

^1^Department of Physics, University of South Florida, Tampa, Florida, USA

^2^Department of Molecular Medicine, USF Health, Tampa, Florida, USA

**Deep-blue Autofluorescence is unaffected by Extensive Dialysis**

To exclude contributions from small molecule contaminants in the Worthington hewL stock, we dialyzed lysozyme in 2 liters of water with four changes during a 2 day period. We then measured the dbAF fluorescence spectra of the dialyzed and of freshly dissolved hewL at nearly identical concentrations.


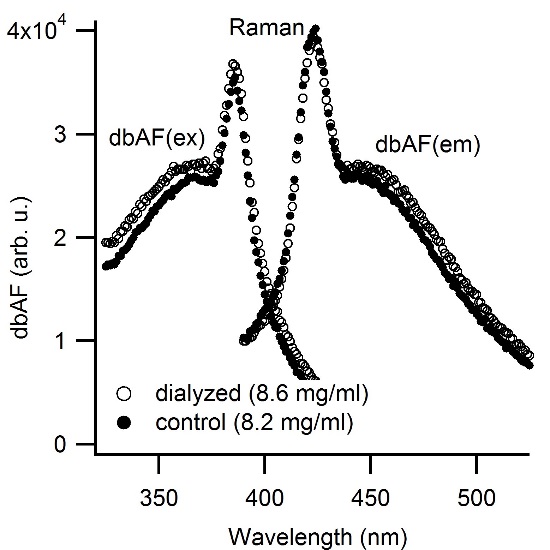


**Figure S1**: Comparison of dbAF spectra for hen egg-white lysozyme (hewl) in pH 7 buffer before (8.2 mg/ml sample) and after (8.6 mg/ml sample) extensive dialysis against water. Contributions from buffer Raman peak were not removed from these spectra.

**Deep-blue Autofluorescence from Amino Acid Crystals**

We investigated whether crystals of amino acids, similar to protein crystals, displayed dbAF emission. Shown below are brightfield and fluorescence images of eight different amino acid crystals obtained using an Olympus IX-70 inverted microscope with an Olympus 40x objective (UApo WI, 1.15 NA) and an Andor Ixon camera (DV-885K; settings: -60 °C cooling, 3.2× preamp. gain, no EM gain). Fluorescence illumination was provided by a 385 nm LED (Thorlabs M385L2, 750 mA drive current) and a standard DAPI fluorescence filter cube (Olympus UM31000: AT350/50 nm ex, 400 dclp dichroic and D460/50 em).


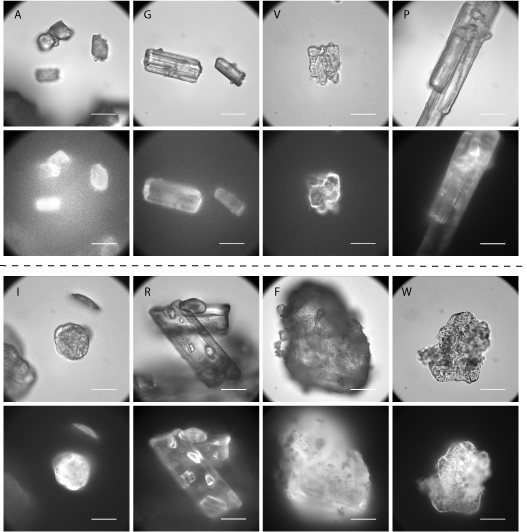


**Figure S2**: Brightfield and epifluorescence images of eight different amino acid crystals. Amino acids are identified by their single-letter code added to their brightfield images. Exposure times for epifluorescence images decreased from 20 s (A) to 5 s (G, V, P, R), 2 s (I) and 0.5 s (W, F). For display, grayscales of fluorescence intensities were adjusted (undistorted relative fluorescence intensities are shown in Fig. 3F of the main text). Amino acid stock was identical to that used for measuring dbAF spectra in solution. Scale bar in all images: 50 μm.

**Molar Extinction Coefficient and Quantum Yield Determinations for Beta-Lactoglobulin (BLG) and Proline (Pro)**

To determine the molar extinction coefficient ε and quantum yield QY for BLG and Proline we measured the absorbance, fluorescence excitation and emission spectra for a series of BLG and Proline concentrations. Typical spectra for BLG are shown in Fig. S3A. Fig. 4D in the main text shows a corresponding series for Proline. The molar extinction coefficient ε was determined in pH 7 buffer (20 mM HEPES) from a plot of the absorbance measured at 357 nm

A B C


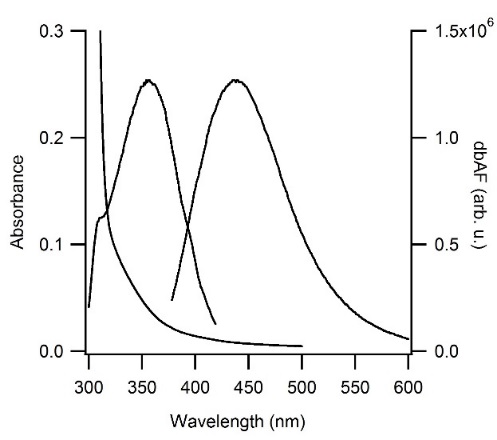

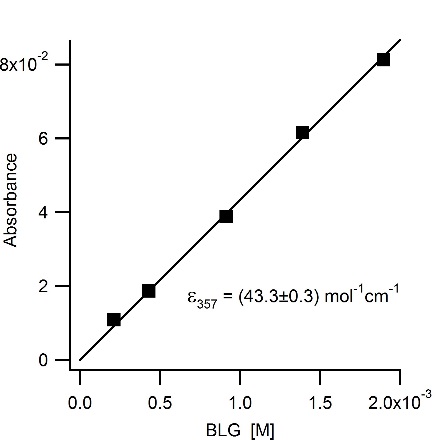

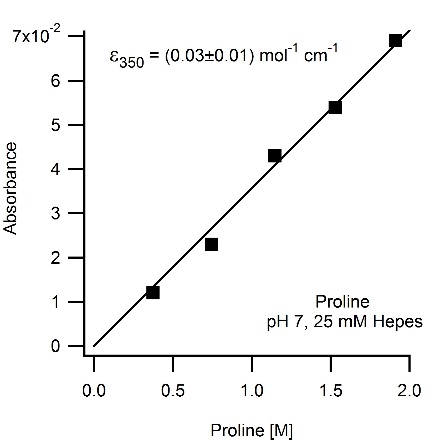


D E


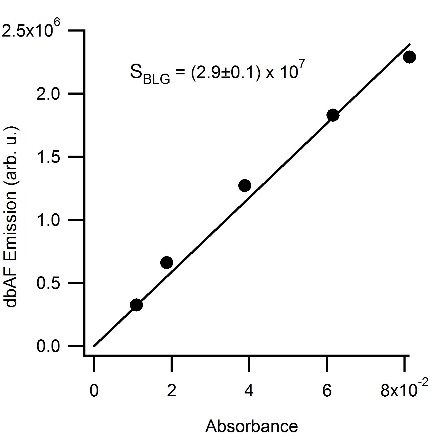

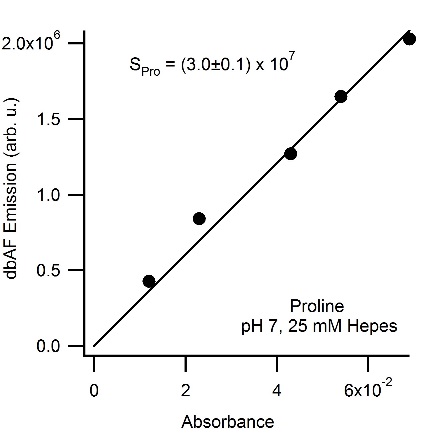


**Figure S3**: (**A**) Absorbance, fluorescence excitation and emission spectra for 16.8 mg/ml of BLG (Sigma) at pH 7. Plots of the absorbance vs. molar concentration for (**B**) BLG and (**C**) proline. Plot of fluorescence peak emission intensity vs. absorbance for (**D**) BLG and (**E**) proline used to extract the slopes S_BLG_ and S_Pro_, respectively

for BLG (350 nm for Proline) against its molar concentration. For QY, dbAF was extracted from the slope S_X_ of the peak emission intensity (437 nm for BLG, 428 nm for Proline) plotted against absorbance. The corresponding quantum yield QY was determined from comparison with the known quantum yield of quinine sulfate (0.54) and the experimentally determined slope S_QS_ of quinine sulfate fluorescence emission vs. absorbance measured in 0.1 M sulfuric acid with the same instrument setting (see Eqn. 1 in the Materials and Methods section).
